# A New Oviraptorosaur (Dinosauria: Oviraptorosauria) from the Late Cretaceous of Southern China and Its Paleoecological Implications

**DOI:** 10.1371/journal.pone.0080557

**Published:** 2013-11-27

**Authors:** Junchang Lü, Laiping Yi, Hui Zhong, Xuefang Wei

**Affiliations:** 1 Institute of Geology, Chinese Academy of Geological Sciences, Beijing, China; 2 Ganzhou Museum of Natural History, Ganzhou, Jiangxi Province, China; 3 Cores and Samples Center of Land and Resources, China Geological Survey, Sanhe, Hebei Province, China; University of Pennsylvania, United States of America

## Abstract

A new oviraptorosaur *Nankangia jiangxiensis* gen. et sp. nov. is described on the basis of a partial postcranial skeleton with a partial lower jaw collected from the Upper Cretaceous Nanxiong Formation of Ganzhou, in Jiangxi Province of southern China. The new taxon is diagnosed by: (1) a mandibular symphysis that is not turned down; (2) neural spines of the cranial caudal vertebrae that are wider transversely than anteroposteriorly, forming a large posterior fossa with rugose central areas; (3) a femoral neck extending at an angle of about 90 to the shaft; and (4) a ratio of femur to tibia length of 0.95. Phylogenetic analysis recovers *Nankangia* as basal to the oviraptorid *Yulong*, but more derived than *Caenagnathus*, which also has a mandibular symphysis that is not turned down. The coexistence of *Nankangia jiangxiensis*, *Ganzhousaurus nankangensis, Jiangxisaurus ganzhouensis*, an unnamed oviraptorid from Nanxiong Basin and *Banji long* suggests that they occupied distinct ecological niches. *Nankangia* may have been more herbivorous than carnivorous.

## Introduction

**Figure 1:**
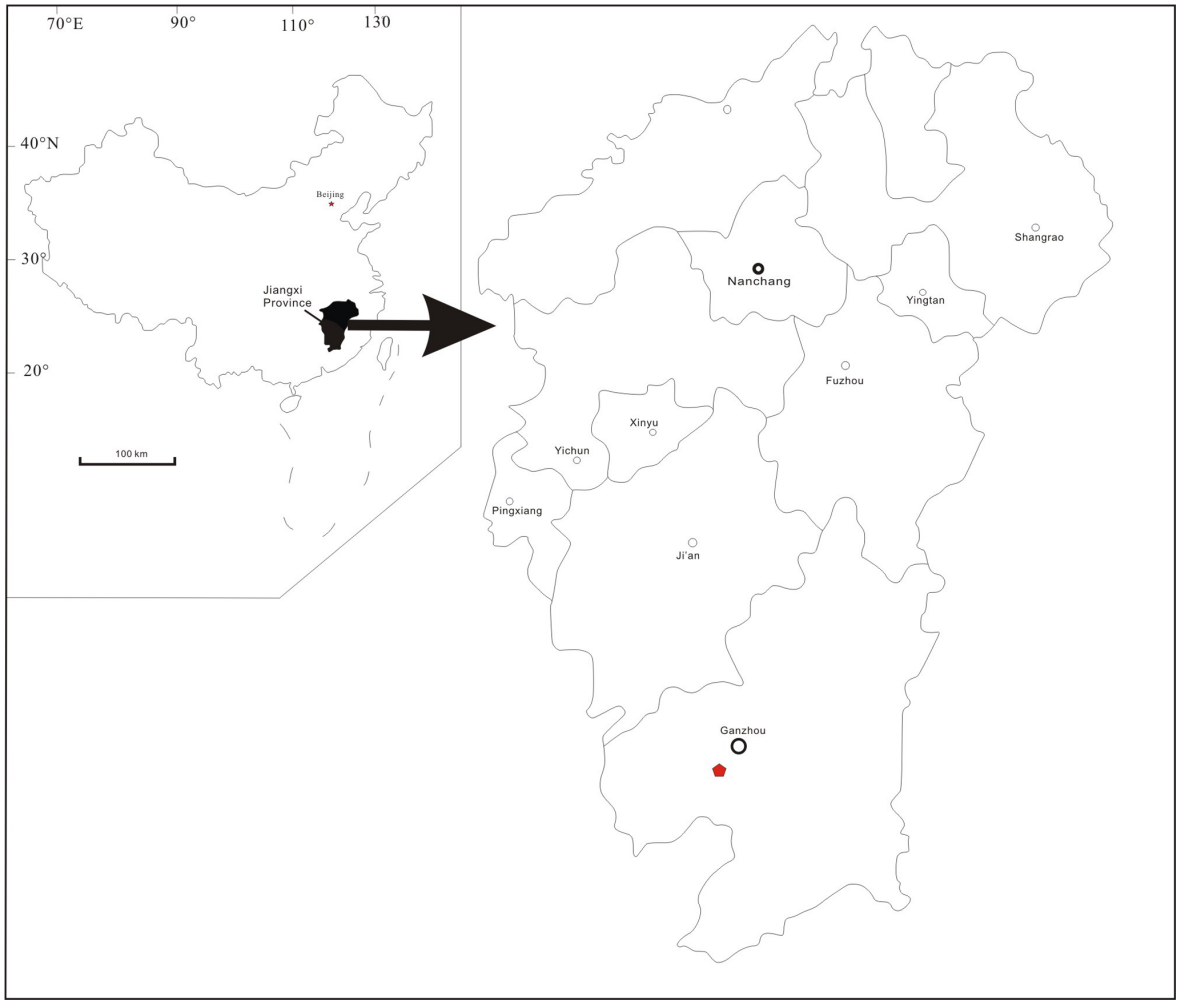
Map of the fossil locality, near Ganzhou, Jiangxi Province, southern China. The solid pentagon represents the fossil site.

Oviraptorids are specialized non-avian theropod dinosaurs that have been reported from Mongolia [1]–[12] and China [13]–[23]. The closely related caenagnathids are best known from North America 31 [24]–[29]. Oviraptorosaurs are known from many regions of China [13]–[18], [21]–[23], [30], [31]. Recently, many oviraptorosaur nests and eggs, along with at least ten oviraptorosaur skeletons buried with the eggs (All oviraptorosaur skeletons that have been published from the Ganzhou area thus far are incomplete because, during quarrying of the rocks for building material, most specimens are destroyed) has made the Ganzhou district one of the most productive localities for oviraptorosaur fossils. Here we report on a new oviraptorosaur, *Nankangia jiangxiensis* gen. et sp. nov., from the Upper Cretaceous Nanxiong Formation of Nankang City, Jiangxi Province (Figure 1). Its discovery plays a key role in our understanding of the distribution and paleoecology of oviraptorosaurian dinosaurs.

## Methods

This study was based on specimen GMNH F10003, housed in the Ganzhou Museum of Natural History, in Ganzhou City, Jiangxi Province. The specimen was donated to the Ganzhou Museum of Natural History three years ago by a local farmer. Two of the authors (L. Yi and H. Zhong) from the Ganzhou Museum of Natural History joined the study of the specimen, and the senior author (J. Lü) obtained permission from the museum to access the collections.

To evaluate the systematic position of *Nankangia* within Oviraptorosauria, 182 characters were scored for 20 taxa (data matrix based on [31]) for phylogenetic analysis. The analysis was performed using the branch-and-bound search algorithm of PAUP* 4.0 b10 [32]. Running the entire data matrix of 20 taxa (including *Herrerasaurus*, *Archaeopteryx*, and *Velociraptor* as outgroups, plus most known oviraptorosaurs) and 182 characters produced two most parsimonious trees, each with a length of 370 steps (consistency index of 0.58, homoplasy index of 0.42, and retention index of 0.69).

### Nomenclatural Acts

The electronic edition of this article conforms to the requirements of the amended International Code of Zoological Nomenclature, and hence the new names contained herein are available under that Code from the electronic edition of this article. This published work and the nomenclatural acts it contains have been registered in ZooBank, the online registration system for the ICZN. The ZooBank LSIDs (Life Science Identifiers) can be resolved and the associated information viewed through any standard web browser by appending the LSID to the prefix “http://zoobank.org/”. The LSID for this publication is: urn: lsid: zoobank.org: pub: C9F22590-8438-427A-9E46-BE2A8CF7FCD2. The electronic edition of this work was published in a journal with an ISSN, and has been archived and is available from the following digital repositories: PubMed Central, LOCKSS.

## Results

### Systematic Paleontology

**Figure 2:**
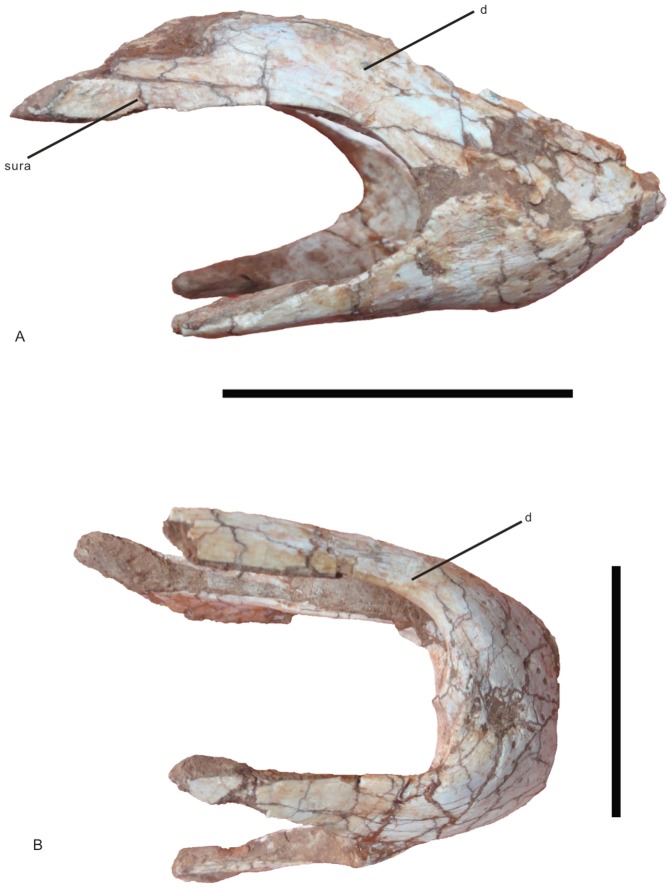
Lower jaw of *Nankangia* (GMNH F10003) in right lateral (A) and ventral (B) views. Abbreviations: d., dentary; sura., surangular. Scale bars  = 5 cm.

**Figure 3:**
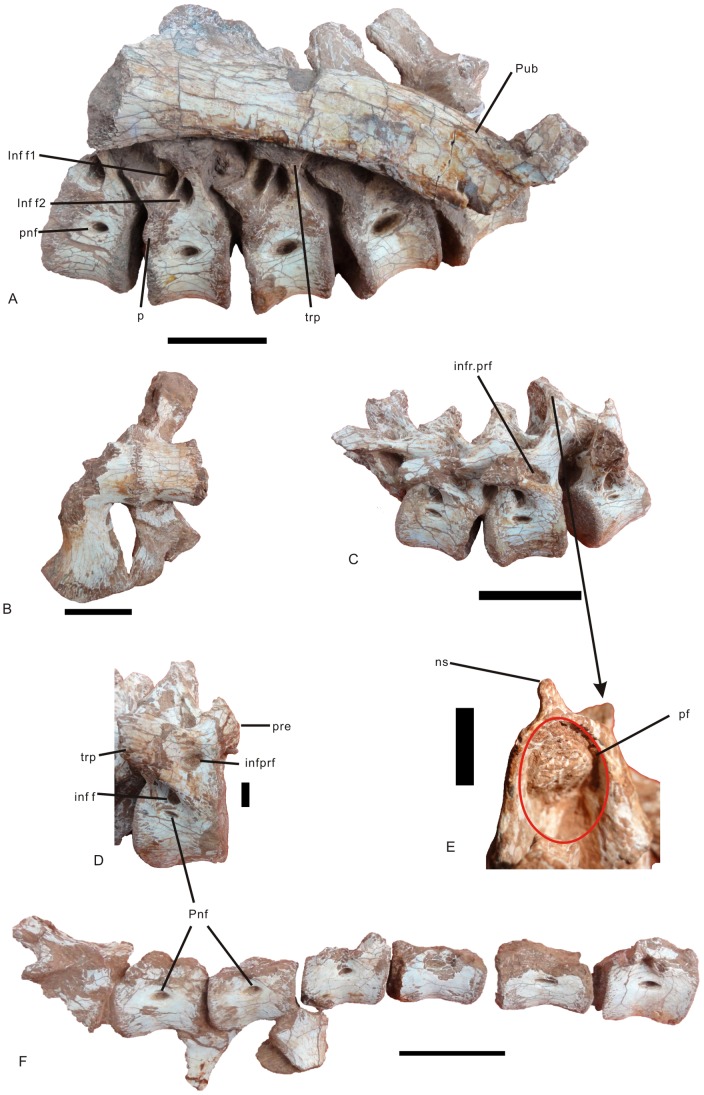
Vertebral column of *Nankangia* (GMNH F10003). **A**, Dorsal vertebrae in left lateral view (partially obscured by adhered pubis); **B**, Sacral vertebrae in ventral view; **C**, Cranial caudal vertebrae in right lateral view; **D**, Detail of cranial-most caudal vertebra in right lateral view; **E**, Cranial caudal neural spine in posterior view; **F**, Middle and posterior caudal vertebrae in right lateral view. Abbreviations: inf f., infradiapophyseal fossa; infr f1., infradiapophyseal fossa 1; infr f2., infradiapophyseal fossa 2;, infr. prf., infraprezygapophyseal fossa; ns., neural spine; p., parapophysis; pf., posterior fossa on the neural spine; pnf., pneumatic fossa; pre., prezygapophysis; pub., pubis; trp., transverse process. Scale bars  = 1 cm in **D**, **E**; others are 5 cm.

**Figure 4:**
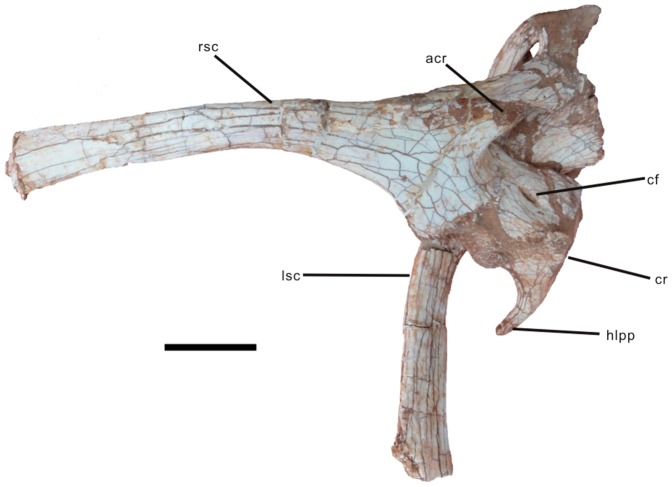
Scapulocoracoids of *Nankangia* (GMNH F10003) in lateral (right scapulocoracoid) and medial (left scapulocoracoid) views. Abbreviations: acr., acromion of the scapula; cf., coracoid foramen; cr., coracoid; hlpp., horn-like posteroventral process; lsc., left scapula; rsc., right scapula. Scale bar  = 5 cm.

**Figure 5:**
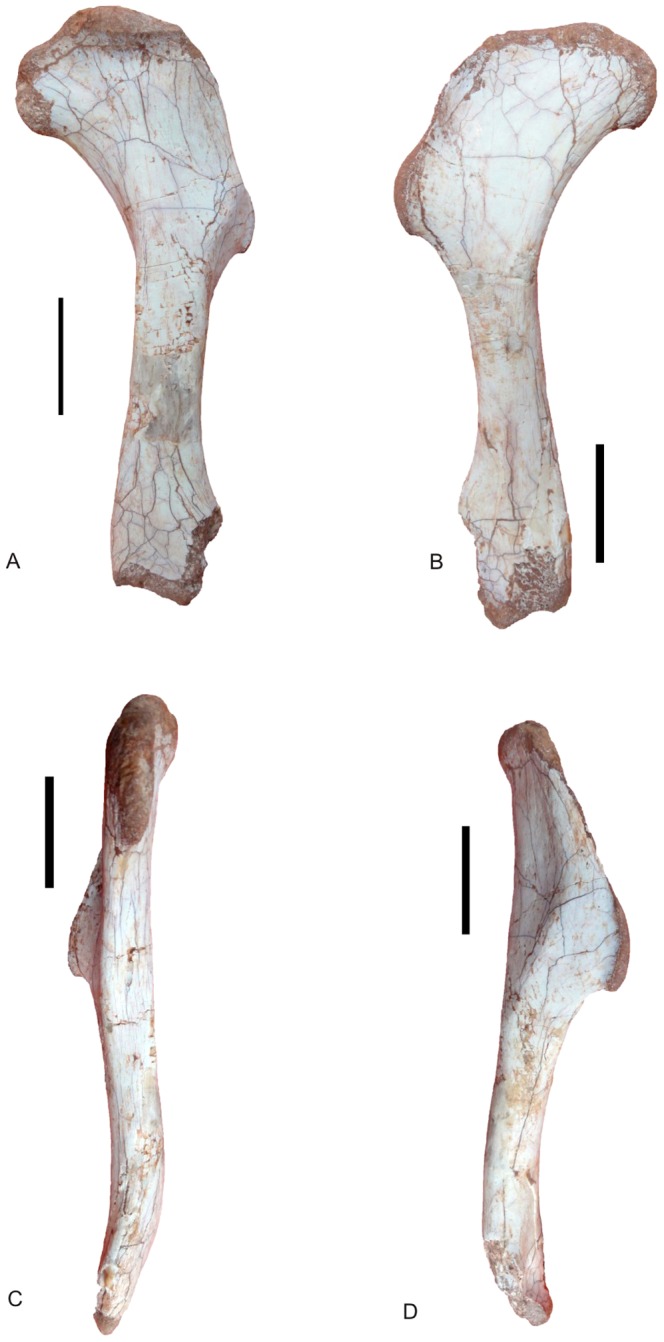
Right humerus of *Nankangia* (GMNH F10003), in posterior (A), anterior (B), medial (C), and lateral (D) views. Scale bar  = 5 cm.

**Figure 6:**
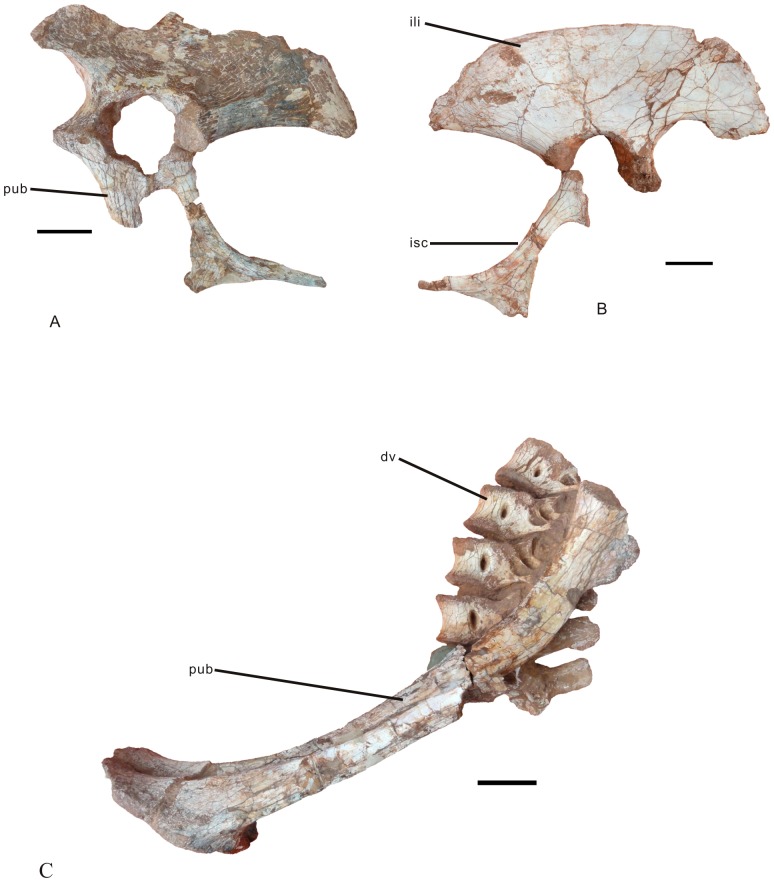
Pelvic girdle of *Nankangia* (GMNH F10003). **A**, Partial left pelvis (missing dorsal edge of ilium and most of pubis) in lateral view. **B**, Partial right pelvis (missing pubis) in lateral view. **C**, Pubes in left lateral view. Abbreviations: dv., dorsal vertebrae; ili., ilium; isc., ischium; pub., pubis. Scale bar  = 5 cm.

**Figure 7:**
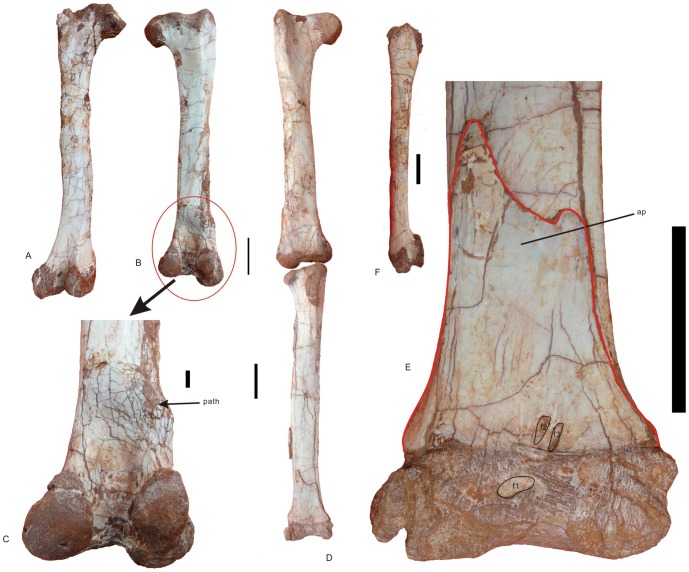
Femur, tibia, and proximal tarsals of *Nankangia* (GMNH F10003). **A**, Left femur in posterior view; **B**, Right femur in posterior view; **C**, Close up of the distal portion of the right femur in posterior view, showing the pathological area; **D**, Right femur and tibia in anterior view; **E**, Close-up of the distal portion of the tibia, the astragalus, and the calcaneum in anterior view; **F**, Left femur in lateral view. Abbreviations: ap., ascending process of astragalus; cal., calcaneum; f1–f3, fossae 1–3; path., pathological areas. Scale bar  = 1 cm in **C**; 5 cm in other images.

Theropoda Marsh, 1881 [33].
Oviraptorosauria Barsbold, 1976 [2].
*Nankangia* gen. nov. (Figures 2, 3, 4, 5, 6, 7).

#### Etymology

*Nankangia* refers to the Chinese administrative unit Nankang City in Jiangxi Province.

#### Type species

*Nankangia jiangxiensis* gen. et sp. nov. urn:lsid:zoobank.org:act: D3C8151C-970B-4944-AA4C-E913E73F64D8.

#### Diagnosis

As for the type and only known species.

#### Etymology

The specific name refers to Jiangxi Province, where the holotype site in Nankang City is located.

#### Holotype

The holotype specimen (GMNH F10003) includes a partial lower jaw, five dorsal vertebrae, one and one-half sacral vertebrae, nine complete and two partial caudal vertebrae, both scapulocoracoids, incomplete furcula, a nearly complete right humerus, complete right ilium, most of the left ilium, complete right pubis and most of the left pubis, complete right ischium and a partial left ischium, both femora, right tibia, and some dorsal ribs. All specimens are housed at the Ganzhou Museum of Natural History, Ganzhou City of Jiangxi Province.

#### Type locality and horizon

Longling of Nankang, Ganzhou City; Nanxiong Formation (Upper Cretaceous) [34].

#### Diagnosis

*Nankangia* is distinguished by the following combination of characters: (1) the rostral end of the mandibular symphyseal region is not downturned (shared with caenagnathids, *Incisivosaurus*, *Luoyanggia* and *Ganzhousaurus*); (2) two infradiapophyseal fossae on the ventral surface near the base of the transverse process of the dorsal vertebrae; (3) pneumatic fossae on the sacral vertebrae slit-like; (4) neural spines of the cranial caudal vertebrae wider transversely than anteroposteriorly, forming a large posterior fossa with a rugose central area; (5) large fossa on the anterior surface (infraprezygapophyseal fossa) and another (infradiapophyseal fossa) on the ventral surface of the base of the transverse process of the cranial caudals; (6) femur longer than ilium (shared with *Yulong* and *Khaan*); (7) ratio of height to length of ilium 0.36; (8) femoral neck extending dorsomedially at about an angle of 90 to the shaft; (9) femur and tibia approximately the same length.

### Description

#### Lower jaw

Both sides of the rostral part are preserved (Figure 2). In lateral view, the rostral end of the jaw is not downturned and the ventral margin of the dentary is nearly straight (Fig. 2A). Posteriorly, the dentary is divided into two branches that extend posterodorsally and posteroventrally, forming the anterior margin of the external mandibular fenestra. Many small nutrient openings are irregularly distributed on the lateral surface of the mandibular symphyseal region. In dorsal view, the cranial margin of the rostral end of the lower jaw is nearly straight; thus the mandibular symphyseal region is U-shaped, similar to that in *Khaan mckennai* and other oviraptorosaurs [12], but different from that of *Luoyanggia liudianensis*, where it is V-shaped [21]. The dorsal surface of the mandibular symphyseal region is smooth with several foramina along the margin of the jaw. The mandibular symphyseal suture is nearly straight. There is a distinct opening on the suture near the rostral end of the lower jaw. The margins of the rostral end are sharp. In posterior view, the sutural portion is convex, forming a distinct ridge. In ventral view, the symphyseal region is also U-shaped (Fig. 2B).

#### Dorsal vertebrae

(Fig. 3 A; see Table S1). Five dorsal vertebrae are preserved. According to the position of the parapophysis, these vertebrae are mid-dorsal vertebrae. But their exact positions in the vertebral column are not clear, and thus these five dorsal vertebrae are referred to here as dorsal 1 to dorsal 5. The anterior articular end of the centrum is much more concave than the posterior articular end, which is moderately concave. The articular end is higher than wide and nearly rectangular. The five dorsal vertebrae are tightly articulated, and their lateral sides are covered by a portion of pubis on the left side and dorsal ribs on the right side. Thus, the detailed structure of the neural arches is not clear. The five articulated dorsal vertebrae curve dorsally. The position of the parapophysis moves upwards from the anterodorsal corner of the centrum on dorsal 1 to the neural arch on dorsal 5. The lengths of the centra increase from the cranial to the caudal vertebrae, as do the lengths of the lateral pneumatic fossae. A weak ventral ridge is present on the first two dorsal vertebrae. The ventral surfaces of the more caudal vertebrae become round. The facet of the parapophysis on dorsal 2 extends across the suture of the neural arch and the centrum, although its details are not clear. The facet of the parapophysis is concave and elongated, and its long axis is nearly vertical. The neural spine extends perpendicular to the centrum. Two distinct fossae (infradiapophyseal fossa: Infr f1 and Infr f2) are present on the lateral surface near the base of the transverse process (Fig. 3A). These two fossae are demarcated by three laminae: the anterior lamina originates from the posterolateral margin of the prezygapophysis and extends to the anteroventral surface of the transverse process; the middle lamina originates from the dorsal margin of the parapophysis and extends to the ventral surface of the transverse process; and the last lamina extends from the posterolateral margin of the centrum to the ventral side of the transverse process.

#### Sacral vertebrae

(Fig. 3B; see Table S1 for measurements). There are only one and one half sacral vertebrae preserved. They represent the posteriormost two sacrals. The transverse processes are stout, with expanded distal ends that are fused together. The ventral surfaces of the centra are smooth. The suture between them is visible. The ventral surface of the last sacral centrum is rounded with a slight, shallow groove. The transverse process of this vertebra extends posterolaterally beyond the posterior articular end of the centrum. The posterior articular end of the posteriormost sacral vertebra is moderately concave and slightly wider than high. The neural canal is large and wider than high. The lateral surface of the last sacral vertebra is smooth with a small pneumatic fossa.

#### Caudal vertebrae

(Fig. 3C; see Table S1 for measurements). Nine complete and two partial caudal vertebrae are preserved. A set of three and one half relatively large, articulated caudal vertebrae are cranial caudals. Another two and one half smaller vertebrae are distal caudals, and four isolated vertebrae belong to the middle portion of the tail.

The anterior articular ends of all completely preserved caudal vertebrae are moderately concave and their posterior articular ends are slightly concave. Because it is difficult to determine the exact positions of these vertebrae, they are here referred to as caudal 1 to caudal 9. Caudal 1 is relatively stout. The anterior articular end of its centrum is concave, although the margins are weathered and the articular end is not as concave as on the posterior caudals. The anterior articular end is circular. The ventral surface of the centrum is convex. The facet of the prezygapophysis faces anteromedially, and the prezygapophysis extends forward beyond the anterior articular end in lateral view. In anterior view, the neural canal is rectangular and higher than wide. The postzygapophysis does not extend beyond the posterior articular end. The pneumatic fossa is slit-like and smaller than those on the posterior caudal vertebrae (Fig. 3C). The transverse process extends posterolaterally. There is a distinct fossa (the infraprezygapophyseal fossa) on the lateral surface near the junction of the prezygapophysis and transverse process (Fig. 3D). Another triangular fossa (the infradiapophyseal fossa) is situated on the ventral surface near the base of the transverse process. This large fossa is demarcated by strong laminae known as the anterior and posterior centrodiapophyseal laminae in sauropod dinosaurs [35]. The neural spine is wider transversely than long anteroposteriorly. A distinct median vertical ridge (a structure that would be called a prespinal lamina in a sauropod) is present on the anterior surface of the neural spine, but this ridge disappears before reaching the dorsal margin of the neural canal. The neural spine extends posterodorsally and is triangular in lateral view. The posterior surface of the neural spine between the postzygapophyses is strongly concave, forming a large posterior fossa with a rugose area (Fig. 3E, pf) for the attachment of interspinal ligaments. The structures of the next two caudal vertebrae are similar to the first one, except for the pneumatic fossae and infraprezygapophyseal fossa, which become larger, and the infradiapophyseal fossa, which becomes smaller. The broken surfaces of the neural arches show that these structures are highly pneumatic.

The remaining caudal vertebrae are smaller than the first three caudals (Fig. 3F). Their centra have circular, concave anterior articular ends. They have distinct ventral grooves for contact with the hemal arches. A weak ventral groove is present on these distal caudal vertebrae. The prezyzapophysis overlaps a small part of the preceding vertebra. The hemal arch is wider than long. The hemal arch facets are distinct.

#### Scapulocoracoid

(Figure 4; see Table S2 for measurements). Both the right and left scapulocoracoids are firmly attached to each other. The scapula and coracoid are highly fused and their sutures are not clear. The shaft of the scapula is narrow and curved medially. Its dorsal surface is weakly convex and its ventral surface is slightly concave in lateral view. Its dorsal margin is moderately thicker than its ventral margin. The distal end of the scapula is slightly expanded. The proximal end is much more expanded than the distal end. The acromion is very well developed. It is long and projects anteriorly. In lateral view, the ventral margin of the acromion smoothly transitions to the anterior surface of the proximal end of the scapula, forming a U-shaped configuration. The glenoid cavity faces posteroventrally. The coracoid is sutured with the scapula along a straight margin. Its anterior margin is missing. Its ventral margin is convex and its posterior margin is concave, forming a horn-like posteroventral process, which extends posteroventrally beyond the glenoid. The lateral surface of the coracoid is strongly convex with a distinct biceps tubercle near the center of the coracoid. The coracoid foramen is slit-like and its long axis nearly perpendicular to the scapula-coracoid suture. It is located anterodorsal to the biceps tubercle. The medial surface of the coracoid is strongly concave anterior to the posterior margin of the coracoid.

#### Furcula

The bone is broken, and only parts of it are in contact with the acromion.

#### Humerus

(Figure 5; see Table S2 for measurements). The right humerus is almost completely preserved, missing only a small portion of its distal end. It is slightly twisted. The deltopectoral crest is low, and its margin becomes thicker than the lateral margin of the proximal end of the humerus. The anterolateral surface of the proximal end of the humerus is concave. The posterior surface of the humerus is slightly convex. The lateral margin of the deltopectoral crest is straight. There is a distinct ridge that originates from the level of the distal margin of the deltopectoral crest nearly parallel to the apex of the deltopectoral crest, which is separated from it by about 2.5 cm. The apex of the deltopectoral crest is located on the proximal one-third of the shaft, which is different from the condition in some oviraptorids, where the apex of the deltopectoral crest is situated just proximal to the mid-length of the shaft [3]. The internal tuberosity expands medially. A distinct concave surface separates the internal tuberosity from the humeral head on the posterior surface. The humeral head is convex posteriorly but its opposite side is nearly flat. A portion of the distal end of the humerus is missing, thus the detailed structure of the condyles is not clear. The area of the olecranon fossa is flat on the posterior surface of the distal articular end of the humerus. There is a distinctly concave, round area near the middle portion of the distal end.

#### Ilium

(Fig. 6A, B; see Table S3 for measurements). The right ilium is complete (Fig. 6B) and the left ilium (Fig. 6A) is missing its dorsal margin. The dorsal margin of the ilium is arched (Fig. 6B). In dorsal view, the preacetabular process extends anterolaterally, whereas the postacetabular process extends posteromedially; thus the ilium is weakly sigmoid in dorsal view. Although the ilia are not preserved together, the position of the medially curved central portion of the ilium may indicate that the dorsal margins of the opposing iliac blades were close to or even contacted each other along their medial sections. The preacetabular process of the ilium is slightly longer than the postacetabular process. The central portion of the lateral surface of the ilium is concave. The lateral surface of the postacetabular process is convex and that of the preacetabular process is nearly flat. The postacetabular process is much thicker than the preacetabular process. The preacetabular process is thin and plate-like, and its ventral margin is weakly expanded ventrally below the level of the dorsal acetabular margin. The ventral margin of the preacetabular process is mediolaterally wide and there is a distinct medial margin, which forms a shallow fossa. This cuppedicus fossa is near the base of the preacetabular process. The anteroventral extension of the preacetabular process is round. The postacetabular process narrows posteriorly and is pointed. The brevis fossa is short and wide on the ventral surface of the postacetabular process. The pubic peduncle is much deeper than the ischial peduncle, which is different from other oviraptorids where the pubic peduncle is as deep as the ischial peduncle [3]. The pubic peduncle is much longer anteroposteriorly than the ischial peduncle. The articular surface of the ischial peduncle is much smaller than that of the pubic peduncle. The pubis is fused with the ilium but the ischium is not fused. The articular surface on the ischial peduncle for the ischium is small, triangular, and located posteroventrally. The antitrochanter is located on the lateral surface near the ischial peduncle. There is no supracetabular crest. The acetabulum is wider on the lateral surface than the medial surface.

The medial surface of the ilium is slightly concave dorsoventrally. There are two short, distinct ridges extending dorsally above the middle dorsal margin of the acetabulum.

#### Pubis

(Fig. 6C; see Table S4 for measurements). The right pubis is complete whereas the left pubis is missing its middle portion. The pubis is oriented vertically and its shaft is weakly concave anteriorly. The proximal end of the pubis forms the anteroventral margin of the acetabulum. The distal ends of both pubes are fused into a pubic boot. The anterior process of the pubic boot is longer than the posterior process. The ventral surface of the pubic boot is flat. Although the pubic apron is not well preserved, broken surfaces indicate that the apron occupied about two to thirds of the length of the pubis.

#### Ischium

(Fig. 6A, 6B; see Table S4 for measurements). The right ischium is complete. The left ischium is missing a small portion of the middle of its shaft (Fig. 6A). The ischium has a very well developed, triangular obturator process whose tip is 15 cm away from the proximal end of the ischium (Fig. 6B). The articular surface for the ischial peduncle of the ilium is circular whereas the articular end for the pubis is elongated. The medial surface of the ischium is flat and its lateral surface, except for the obturator process, is slightly convex. The lateral surface of the obturator process is concave. The posterior margin of the ischium is thick, round, and distinctly concave. The anterior margin of the ischium is thin. There is no evidence for the fusion of the distal ends of the ischia.

#### Femur

Both femora are well preserved (Fig. 7A–D, F; see Table S5 for measurements). A swollen area near the distal end of the right femur is probably pathological in origin. This area is about 9 cm away from the distal end of the femur. The right femur is shorter than the left femur, and this might be caused by this pathology. The femur is longer than the ilium. It is straight in anterior and posterior views but it curves anteriorly in lateral view. The femoral head is more or less oval and the femoral neck is distinct. The femoral neck extends medially at an angle of about 90 to the shaft. In posterior view, the femoral head bears a distinct ridge, which is separated from the femoral neck. There is a distinct foramen on the posterior surface near the middle of the proximal end. This foramen is larger on the left femur than on the right. At the proximal margin, there is a distinctly smooth, shallow area that separates the massive greater trochanter from the femoral neck. The greater trochanter extends anteroposteriorly. The lateral surface of the greater trochanter is smooth and flat. The anterior (lesser) trochanter is finger-like and its dorsal end is well distal to the greater trochanter. The anterior trochanter adheres closely to the greater trochanter but is separated from it by a distinct furrow that opens dorsally. It bears a pronounced ridge on its anterior surface. There is a distinct opening near the base of the anterior trochanter. About 11 cm from the proximal end of the right femur, a suboval area (about 2 cm long) is defined by a shallow groove near the medial margin of the posterior surface of the shaft. This region is not well preserved on the left femur. It is the fourth trochanter. On the distal end of the femur, the medial condyle is larger than the lateral condyle. The lateral condyle extends only slightly distal to the medial condyle, whereas, in oviraptorids, the lateral condyle extends well distal to the medial one [3]. Thus, when seen in anterior view, the distal margin of the femur is nearly horizontal. As in oviraptorids, there is a deep fossa that separates the well-developed tibiofibular crest from the large posterior surface of the medial condyle. In anterior view, the surface of the distal end is nearly flat. A distinct ridge is present on the anteromedial margin and may represent the medial epicondyle. There is a coarse area formed by short ridges and grooves on the distomedial surface of the distal end. This is possibly an anteromedial adductor muscle scar, although it is weak.

#### Tibia

(Fig. 7D; see Table S5 for measurements). The right tibia, along with a small portion of the adherent right fibula, is well-preserved. The tibia is straight and 2 cm longer than the femur. The posterior surface of the shaft is slightly convex whereas its anterior surface is nearly flat. The cnemial crest is strongly developed occupying almost half the entire anteroposterior length of the proximal surface of the tibia. The medial surface of the proximal end is smooth and convex. The proximal articular surface of the tibia is angled obliquely posteriorly. The margins are expanded posteriorly; thus, the proximal end is clearly distinct from the tibial shaft. There is a foramen on the posterior surface just distal to the proximal margin. The fibular condyle of the tibia is oval, with a concave surface for articulation with the fibula. The fibular crest is short, extending for about 7 cm along the anterolateral margin of the proximal portion of the tibia. The distal end of the tibia is slightly expanded.

#### Tarsals

The proximal tarsals (astragalus and calcaneum) are not fused but are tightly applied to each other and to the tibia. The calcaneum is small and disc-like. Its lateral surface for contact with the distal end of the fibula is concave. The astragalus is large. Its ascending process is taller than wide (5.5 cm wide, at least 8.5 cm tall). It extends for at least 21% of the length of the anterior surface of the tibia. There is a suboval fossa on the middle of the anterior surface of its articular end (Fig. 7E, f1). Two small, parallel, elongate fossae (Fig. 7E, f, f2) are present near the base of the astragalus; this may be similar to the well-marked central depression in other oviraptorids [3].

### Phylogenetic analysis

**Figure 8:**
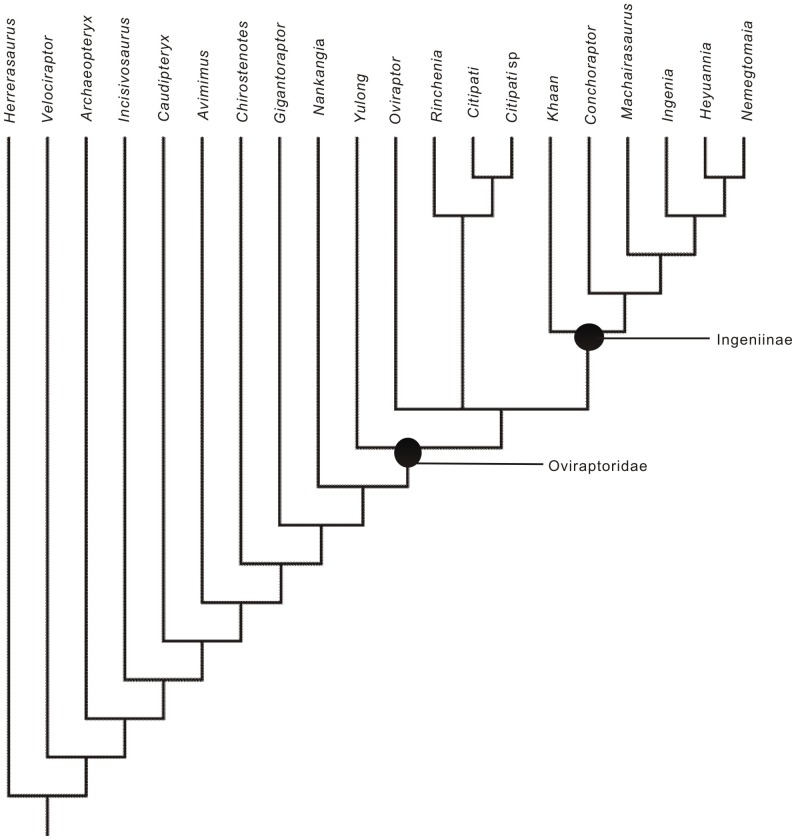
Strict consensus of two most parsimonious trees obtained by PAUP, based on analysis of 20 taxa and 182 characters [31]. Each tree has a length of 370 and was recovered via a branch and bound search (CI = 0.58, HI = 0.42, RI = 0.69).

The phylogenetic analysis, conducted to elucidate the relationships of *Nankangia* within Oviraptorosauria, resulted in two most parsimonious trees, each with a length of 370 steps (consistency index of 0.58, homoplasy index of 0.42, and retention index of 0.69). Figure 8 shows the strict consensus of the two most parsimonious trees (see the supporting information for details: Text S1). The phylogenetic analysis shows that *Nankangia* is basal to the clade (*Yulong* + other Oviraptoridae) but is more derived than *Gigantoraptor*. This is supported by the morphology of the lower jaw: the non-downturned rostral end of the lower jaw in *Nankangia* is short, similar to that of *Yulong*, but is relatively longer in *Gigantoraptor* and *Chirostenotes*. The results support the existence of a monophyletic Oviraptoridae, with two subclades, Oviraptorinae and Ingeniinae. Ingeniinae includes (*Khaan* + (*Conchoraptor* + (*Machairasaurus* + (*Ingenia* + (*Heyuannia* + *Nemegtomaia*))))). The composition of Ingeniinae is unchanged from previous analyses. *Nankangia* shares the following two unambiguous characters with Oviraptoridae: 167 (lateral surface of dentary smooth) and 182 (small process of astragalus protrudes through a circular opening in edge of calcaneum to reach lateral margin of tarsus). It also shares one ambiguous character, 142 (anteroposterior length of pubic peduncle subequal to that of ischial peduncle). *Nankangia* has the following unambiguous synapomorphic characters of Oviraptoridae: 124 (extent of deltopectoral crest [measured from humeral head to apex] ca. 40%–50% of humerus length) and 138 (preacetabular process expanded ventrally well below level of dorsal acetabular margin). It also has the possible autapomorphic characters 75 (downturned symphyseal portion of dentary absent), 120 (acromion projecting anteriorly), 139 (cuppedicus fossa or a wide shelf present on ventral margin of preacetabular process), 143 (dorsoventral extension of pubic peduncle deeper than ischial peduncle), and 150 (anterior and greater trochanters separated).

The results indicate that Ingeniinae is stable as in previous studies [22], [31]. The only difference between the two most parsimonious trees is the position of *Oviraptor*. *Yulong* is recovered as basal to *Oviraptor* (see Fig. 4 of [31]); however, the characters of *Yulong* indicate it is a derived oviraptorid [31]. *Yulong* is here regarded as a member of Oviraptoridae as indicated in the trees. Lastly, although this is not supported by the present phylogenetic analysis, there is some possibility that *Nankangia* may form a clade with *Gigantoraptor* and *Chirostenotes*, pending further study based on more specimens.

## Comparison and Discussion

**Figure 9:**
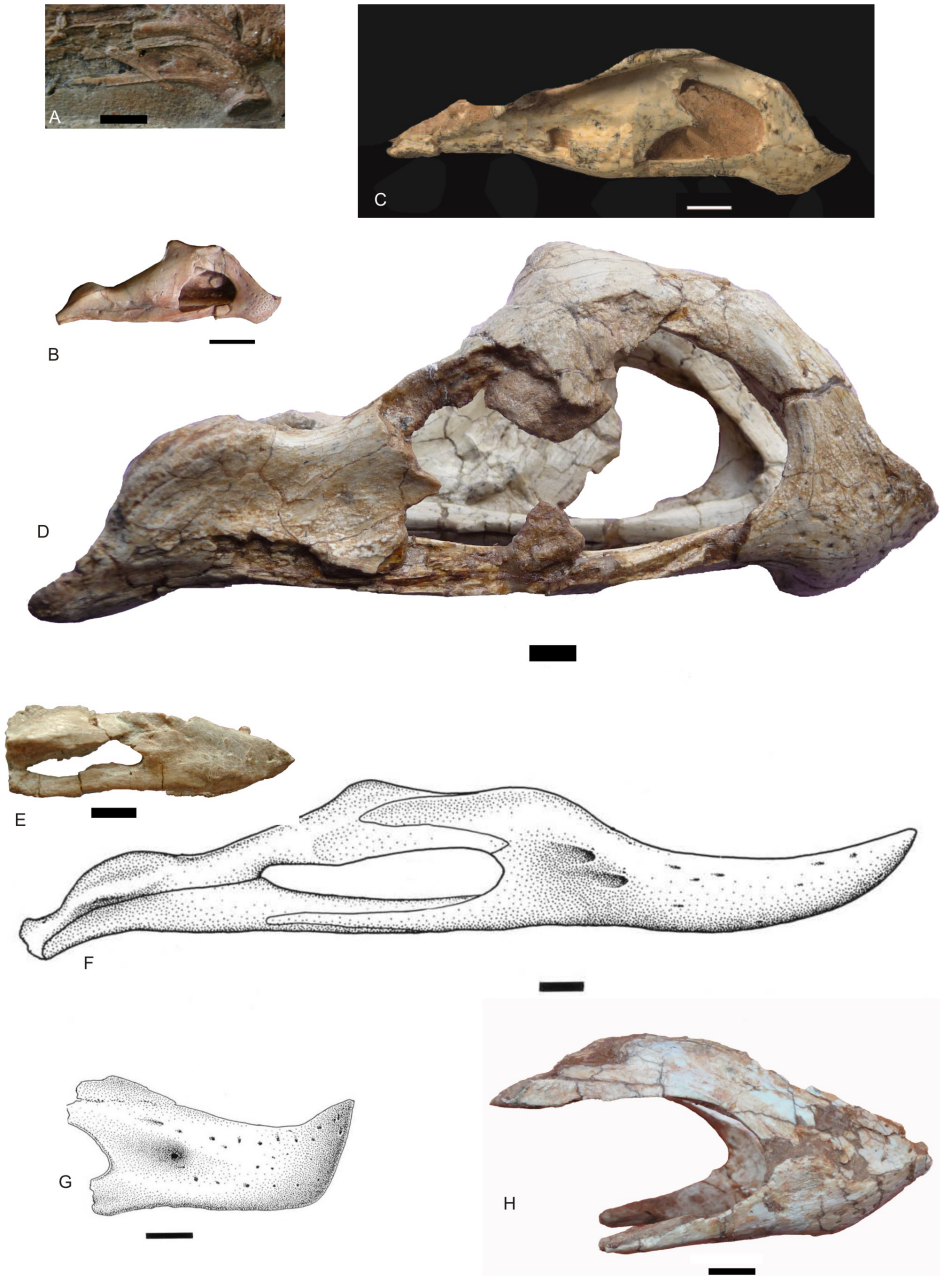
Comparisons of lower jaws (anterior ends) of some oviraptorosaurs. **A**, *Caudipteryx* sp. (IVPP 12430, modified from [14]) (reversed); **B**, *Yulong mini* (HGM 41HIII-0109); **C**, *Khaan mckennai* (IGM 100/973, modified from [12]); **D**, *Nemegtomaia barsboldi* (GIN100/2112); **E**, *Incisivosaurus gauthieri* (IVPP V13326); **F**, *Chirostenotes pergracilis* (CMN 8776: *Caenagnathus collinsi*, from [27]; reversed); **G**, “*Caenagnathus* cf. *sternbergi*” (RTMP 92.36.390, from [27]). **H**, *Nankangia jiangxiensis* gen. nov. (GMNH F10003). Scale bars  = 1 cm.

The rostral portion of the lower jaw in *Nankangia* differs from that of most of derived oviraptorosaurs reported from Asia in which the rostral portion of the lower jaw is preserved (Figure 9). The rostral portion of the mandibular symphysis in *Nankangia* is not downturned and, in lateral view, the ventral margin of the dentary is straight. By contrast, in most derived oviraptorosaurs, such as *Oviraptor* [1], *Citipati*, *Khaan* [8], *Heyuannia* [15], an unnamed oviraptorid from the Nanxiong Basin [16], *Nemegtomaia* [16], *Banji* [23], and *Yulong* [31], the rostral portion of the lower jaw is downturned (Figure 9), and the ventral margin of the dentary is not straight. The structure of the lower jaw is not related to the ontogeny of oviraptorosaurs, because even in juveniles of the oviraptorid *Yulong* [31], the rostral portion of the lower jaw is as strongly downturned as in mature oviraptorids. The structure of the lower jaw, is, however, related to phylogenetic position, because in the primitive *Caudipteryx* [13], [14], the rostral portion of the mandibular symphysis is also downturned. Among the derived oviraptorosaurs, only Caenagnathidae such as *Chirostenotes pergracilis* [36] ( =  *Caenagnathus collinsi* [24], [28]), *Caenagnathus sternbergi* [25], and *Caenagnathasia martinsoni* [27] have a weakly downturned rostral portion of the mandibular symphysis also, in these taxa, the rostral tip of the dentary is turned up (Figure 9). However, as Barsbold [37] pointed out, the mandibular symphysis of caenagnathids is proportionally much longer than in other oviraptorosaurs. Also, in caenagnathids, the rostral portion of the lower jaw (between the anterior margins of the external mandibular fenestrae to the tip) is much shallower and longer than those of Oviraptoridae. Thus, *Nankangia jiangxiensis* gen. et sp. nov. does not appear to belong to Caenagnathidae. The phylogenetic analysis also confirms that the new taxon is more closely related to the oviraptorid *Yulong* than to *Chirostenotes*.

**Figure 10:**
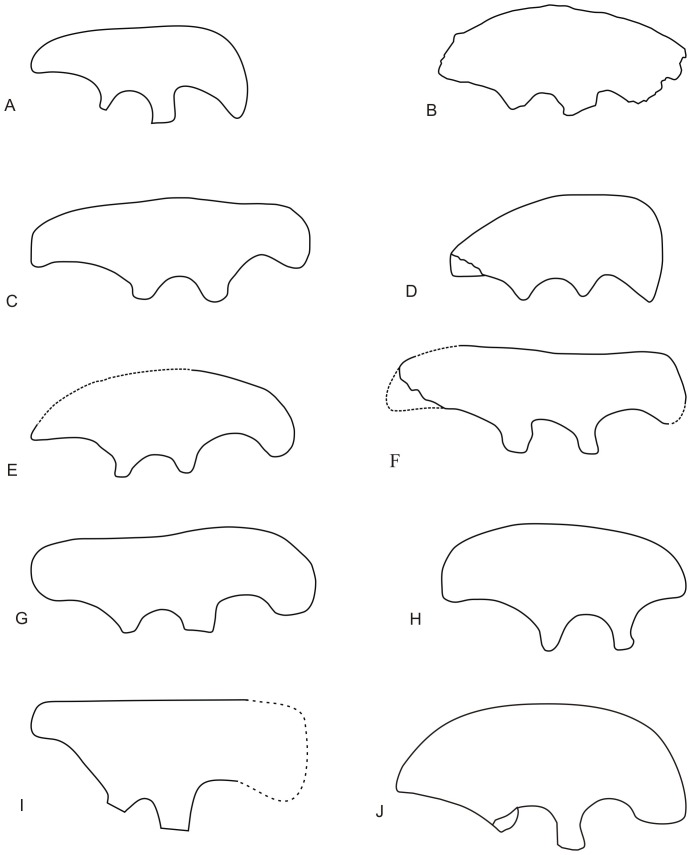
Comparison of ilia among selected oviraptorosaurs in right lateral view. **A**, Left ilium of *Caudipteryx dongi* (reversed); **B**, Left ilium of *Chirostenotes pergracilis* (reversed); **C**, Right ilium of *Ingenia yanshini*; **D**, Right ilium of *Rinchenia mongoliensis*; **E**, Right ilium of *Heyuannia huangi*; **F**, Right ilium of *Nemegtomaia barsboldi*; **G**, Left ilium of *Nomingia gobiensis* (reversed); **H**, Left ilium of *Shixinggia oblita* (reversed); **I**, Left ilium of *Luoyanggia liudianensis* (reversed); **J**, Right ilium of *Nankangia jiangxiensis* gen. et sp. nov. **A**–**H** are modified from [17], **I** is from [21]. No scale.

In *Nankangia*, the dorsal margin of the ilium along the central portion of the blade is arched, which is similar but not identical to the conditions in *Chirostenotes*, *Rinchenia*, *Heyuannia*, and *Shixinggia* (Figure 10). With regard to the arched central portion of the dorsal margin, the ilium of *Nankangia* is most similar to that of *Heyuannia*, but in the latter taxon the pubic peduncle is smaller than the ischial peduncle, where as this is the reverse in *Nankangia*. The ilium of *Nankangia* is distinctly different from those of other oviraptorids, where the dorsal margin of the ilium along the central portion of the blade is either slightly convex, concave, or nearly straight (Figure 10).

Although the dentary of *Luoyanggia* is similar to that of *Nankangia*, the ilium of *Luoyanggia* [21], with a straight dorsal margin, is clearly different from the arched dorsal margin of the ilium in *Nankangia*. Thus these taxa are not synonymous.

Although the dentary of *Nankangia* is similar to that of *Ganzhousaurus*, a taxon was also discovered from the Nanxiong Formation of Ganzhou [38], it differs from *Ganzhousaurus* in the following ways: (1) the dentary of *Ganzhousaurus* appears to have a small “chin” whereas that of *Nankangia* does not; (2) the anterior margin of the dentary is nearly perpendicular to the ventral margin in lateral view in *Ganzhousaurus*, whereas the two margins meet at a more obtuse angle in *Nankangia*; (3) the posterodorsal process of the dentary appears dorsoventrally deeper in *Nankangia* than in *Ganzhousaurus*.

The lower jaw of *Nankangia* is also similar to that of *Jiangxisaurus*, which was also discovered from the same formation of Ganzhou [39]. In ventral view, the cranial portions of the sutures of the lower jaw between these two taxa are different. It is straight in *Jiangxisauurus*, although it may be slightly affected by preservation, it is interlocked in *Nankangia*. In posterior view, the medial margin of the humerus is more curved medially in *Nankangia* than the condition in *Jiangxisaurus*. The detailed comparison is difficult to make, because of lack of the useful corresponding elements between these two taxa.

The rostral portion of the lower jaw is slightly downturned in the basal oviraptorosaur *Incisivosaurus*, which is similar to the condition in *Nankangia*, but *Incisivosaurus* has teeth and its lower jaw is relatively longer than that of *Nankangia*. In these regards, *Incisivosaurus* displays a primitive condition, whereas *Nankangia* is toothless and therefore a more derived oviraptorosaur.

It is difficult to compare *Nankangia* with the recently named Inner Mongolian oviraptorid *Wulatelong* [40], due to the scarcity of corresponding elements between these two taxa. Even the overlapping elements, such as the ilium, femur, and tibia, are not well preserved in *Wulatelong*.

### The paleogeographical distributions of Chinese oviraptorosaurs

Oviraptorosaur fossils have been discovered in northern, central, and southern China. At present, both basal and derived oviraptorosaurs are found in northern China. Primitive oviraptorosaurs are known only from the Lower Cretaceous Jehol Group of northeastern China: *Incisivosaurus* [19], *Ningyuansaurus* [41], *Caudipteryx* [13], [14], *Protarchaeopteryx* [42], and *Similicaudipteryx* [43]. The derived taxa *Machairasaurus leptonychus* [22], *Wulatelong gobiensis* [40], and *Gigantoraptor erlianensis* [20] are from the Upper Cretaceous Bayan Mandahu Formation ( = Wulansuhai Formation [40]) and Iren Dabasu Formation of Inner Mongolia, respectively. Two oviraptorosaurs have been reported from central China: *Luoyanggia* [21] from the upper Lower Cretaceous Haoling Formation [44] and *Yulong mini* [31] from the Upper Cretaceous Qiupa Formation. Including the new genus *Nankangia*, six genera have been discovered from the Late Cretaceous of southern China: *Heyuannia huangi* [15], [16], *Shixinggia oblita* [17], *Banji long* [23], *Ganzhousaurus nankangensis* [38], and *Jiangxisaurus ganzhouensis* [39].

### Palaeoecological implications

It has been suggested that oviraptorosaurs ate eggs, mollusks, nuts, and other hard foods [1], [3], [27], [45], which would have required a strong bite, even in juveniles [31]. However, oviraptorosaurs have also been considered as possible herbivorous theropods [46]. The dentition of *Incisivosaurus* is strongly suggestive of herbivory in this basal oviraptorosaur [19], the gastroliths of *Caudipteryx* [13] and seeds discovered in the body cavity of the only known specimen of *Ningyuansauru*s [41] may also suggest an herbivorous diet. The hind limb proportions are constant in oviraptorids, regardless of absolute body size or ontogenetic stage, which is a pattern seen more commonly in herbivores than in carnivores, and weakly supports the hypothesis that oviraptorids were herbivores rather than active carnivores [31].

The downturned mandibular symphysis of Oviraptorinae and Ingeniinae may have generated a relatively large gape, possibly, though it's also possible that, in life, this “gap” was filled (or partially filled) by a keratinous beak, when these oviraptorids opened their mouths to acquire food. This large gape may have helped these animals to seize relatively large, hard foods such as eggs, nuts, and mollusks. The non-downturned mandibular symphysis of *Nankangia* and *Ganzhousaurus* may indicate that the gape between the upper and lower jaws was smaller in these oviraptorosaurs, which may have been more suitable to procuring and processing small, soft foods such as leaves and seeds. The coeval oviraptorosaur *Banji* and an unnamed oviraptorid from Nanxiong Formation bear a downturned mandibular symphysis, which is different from the non-downturned mandibular symphyses of *Nankangia*, *Ganzhousaurus* and *Jiangxisaurus*. The coexistence of these five oviraptorosaurs taxa implies that they may have occupied different ecological niches. The Nanxiong Formation is perhaps the most diverse oviraptorosaur fauna of any formation in the world. *Nankangia*, *Ganzhousaurus* and *Jiangxisaurus* may have been primarily herbivorous, whereas *Banji* and the unnamed oviraptorid may have been more carnivorous.

## Conclusion

The presence of several unique morphological characters indicates that *Nankangia jiangxiensis* gen. et sp. nov. represents a new taxon of derived oviraptorosaur. *Banji long* [23] from the same locality and same horizon bears a downturned rostral end of the lower jaw, and the structure of its lower jaw is different from those of *Nankangia*, *Ganzhousaurus* and *Jiangxisaurus*. The differing shapes of the lower jaw and the coexistence of *Nankangia*, *Ganzhousaurus*, *Jiangxisaurus*, and *Banji* suggest that they occupied different ecological niches. *Nankangia*, *Ganzhousaurus* and *Jiangxisaurus* may have been primarily herbivorous. The discovery of *Nankangia* plays an important role in improving our understanding of oviraptorosaur distribution and paleoecology.

## Supporting Information

Table S1 Measurements (cm) of the vertebrae of *Nankangia jiangxiensis* gen. et sp. nov. (GMNH F10003).
(PDF)

Table S2 Measurements (cm) of the scapula and humerus of *Nankangia jiangxiensis* gen. et sp. nov. (GMNH F10003).
(PDF)

Table S3 Measurement (cm) of ilium of *Nankangia jiangxiensis* gen. et sp. nov. (GMNH F10003).
(PDF)

Table S4 Measurements (cm) of pubis and ischium of *Nankangia jiangxiensis* gen. et sp. nov. (GMNH F10003).
(PDF)

Table S5 Measurements (cm) of femur and tibia of *Nankangia jiangxiensis* gen. et sp. nov. (GMNH F10003).
(PDF)

Text S1 Phylogenetic analysis.
(PDF)
