# Supplementary material for: A New Oviraptorosaur (Dinosauria: Oviraptorosauria) from the Late Cretaceous of Southern China and Its Paleoecological Implications
Source: PLoS One. 2013 Nov 27;8(11):e80557. doi: 10.1371/journal.pone.0080557 (PMC3842309; doi:10.1371/journal.pone.0080557)
Supplement: Table S1 — Measurements (cm) of the vertebrae of Nankangia jiangxiensis gen. et sp. nov. (GMNH F10003). (PDF) [file pone.0080557.s001.pdf]

Table S1. Measurements (cm) of the vertebrae of *Nankangia jiangxiensis* gen. et sp. nov. (GMNH F10003).

	Length	Anterior articular end		Posterior articular end		Neural canal		Pneumatic fossa length	Transverse width
		width	height	width	height	width	height		
Dorsal 1	3.8	3.1	3.7	3.0	-	1.2	1.3	1.0	-
Dorsal 2	3.7	3.2	-	3.3	-	-	-	1.2	-
Dorsal 3	3.8	3.2	-	3.4	-	-	-	1.3	-
Dorsal 4	3.9	3.6	-	3.6	-	-	-	1.5	-
Dorsal 5	4.0	3.7	-	4.0	-	-	-	1.5	-
Last sacral vertebra	4.3	4.2	-	4.1	3.0	-	-	-	15
Caudal 1	3.8	3.6	3.6	3.5	4.1	0.8	0.9	0.6	8

Caudal 2	4.4	3.7	3.8	3.1	-	-	-	1.0	8.4
Caudal 3	4.2	3.4	-	2.9	-	-	-	0.9	-
Caudal 4	4.1	2.4	2.7	2.4	2.7	-	-	0.8	-
Caudal 5	3.9	3.1	-	2.9	-	-	-	0.9	-
Caudal 6	3.8	2.6	2.5	2.4	2.4	-	-	1.0	-
Caudal 7	4.1	2.6	2.5	2.5	-	-	-	0.9	-
Caudal 8	3.8	2.4	2.6	2.2	-	-	-	0.8	-
Caudal 9	3.7	2.3	-	2.0	-	-	-	0.7	-
