# Supplementary material for: A New Oviraptorosaur (Dinosauria: Oviraptorosauria) from the Late Cretaceous of Southern China and Its Paleoecological Implications
Source: PLoS One. 2013 Nov 27;8(11):e80557. doi: 10.1371/journal.pone.0080557 (PMC3842309; doi:10.1371/journal.pone.0080557)
Supplement: Table S2 — Measurements (cm) of the scapula and humerus of Nankangia jiangxiensis gen. et sp. nov. (GMNH F10003). (PDF) [file pone.0080557.s002.pdf]

Table S2. Measurements (cm) of the scapula and humerus of *Nankangia jiangxiensis* gen. et sp. nov. (GMNH F10003).

	Length	Width	Proximal end width	Distal end width
Scapula	26 (along the shaft)	2.1 (narrowest)	7.8	3.2
Humerus	24	2.8 (mediolateral); 2.1 (anteroposterior); circumference 9.0	6.9	-
Deltopectoral crest	10.8 (measured from humeral head to apex); 16.0 (from the apex to the distal end of the humerus)	1.8	-	-
