# Supplementary material for: A New Oviraptorosaur (Dinosauria: Oviraptorosauria) from the Late Cretaceous of Southern China and Its Paleoecological Implications
Source: PLoS One. 2013 Nov 27;8(11):e80557. doi: 10.1371/journal.pone.0080557 (PMC3842309; doi:10.1371/journal.pone.0080557)
Supplement: Table S3 — Measurement (cm) of ilium of Nankangia jiangxiensis gen. et sp. nov. (GMNH F10003). (PDF) [file pone.0080557.s003.pdf]

Table S3. Measurement (cm) of ilium of *Nankangia jiangxiensis* gen. et sp. nov. (GMNH F10003).

	Length	Height
Ilium	33 (from the tip of the postacebular process to the anterior margin of the preacetabular process)	12 (from the center of the dorsal margin of the acetabulum to the top of the dorsal margin of the ilium)
Acetabulum	Lateral side 8; medial side 5 (inner margin of the acetabulum)	-
Preacetabular process	17 (measured from center of acetabulum)	-
Postacetabular process	16 (measured from center of the acetabulum)	-
