# Supplementary material for: A New Oviraptorosaur (Dinosauria: Oviraptorosauria) from the Late Cretaceous of Southern China and Its Paleoecological Implications
Source: PLoS One. 2013 Nov 27;8(11):e80557. doi: 10.1371/journal.pone.0080557 (PMC3842309; doi:10.1371/journal.pone.0080557)
Supplement: Table S4 — Measurements (cm) of pubis and ischium of Nankangia jiangxiensis gen. et sp. nov. (GMNH F10003). (PDF) [file pone.0080557.s004.pdf]

Table S4. Measurements (cm) of pubis and ischium of *Nankangia jiangxiensis* gen. et sp. nov. (GMNH F10003).

	Length	Width
Pubis	41 (along the shaft), 38 (directly measured from the proximal end to the distal end)	2 (narrowest part of shaft)
Ischium	21.5 (along the shaft); 20.5 (from proximal end to the distal end)	2.5 (shaft)
Obturator process	8	4.5
