# Supplementary material for: A New Oviraptorosaur (Dinosauria: Oviraptorosauria) from the Late Cretaceous of Southern China and Its Paleoecological Implications
Source: PLoS One. 2013 Nov 27;8(11):e80557. doi: 10.1371/journal.pone.0080557 (PMC3842309; doi:10.1371/journal.pone.0080557)
Supplement: Table S5 — Measurements (cm) of femur and tibia of Nankangia jiangxiensis gen. et sp. nov. (GMNH F10003). (PDF) [file pone.0080557.s005.pdf]

Table S5. Measurements (cm) of femur and tibia of *Nankangia jiangxiensis* gen. et sp. nov. (GMNH F10003).

	L	W	Proximal end		Distal end		
			A	B	A	B	C
Right	35	3.9	9.3	2.6	8.0	4.7	11.0
Left	38	3.9	8.6	2.7	7.6	5.1	11.5
Pathological portion (right)	-	5.2 (A)<br>3.4 (B)	-	-	-	-	-
Tibia	40	4.2	7.0	7.5	6.6	1.5	9.8

Notes: A = mediolaterally; B = anteroposteriorly; C = circumference; L= length; W = width.
