# Supplementary material for: A New Oviraptorosaur (Dinosauria: Oviraptorosauria) from the Late Cretaceous of Southern China and Its Paleoecological Implications
Source: PLoS One. 2013 Nov 27;8(11):e80557. doi: 10.1371/journal.pone.0080557 (PMC3842309; doi:10.1371/journal.pone.0080557)
Supplement: Text S1 — Phylogenetic analysis. (PDF) [file pone.0080557.s006.pdf]

Phylogenetic analysis

1). Analysis protocol

To evaluate the systematic position of *Nankangia* among Oviraptosauria, we added the new taxon to the matrix published by Lü et al. [S1].

Phylogenetic analysis was performed using the branch-and-bound search algorithm of PAUP* 4.0b10 [S2]. Running the whole data matrix of 20 taxa (including *Herrerasaurus*, *Archaeopteryx*, and *Velociraptor* as outgroups, plus 17 oviraptorosaurs as the ingroup) and 182 characters produced two most parsimonious trees, each with a length of 370 steps (consistency index of 0.58, homoplasy index of 0.42, and retention index of 0.69). Supplementary Figure 1 depicts the two most parsimonious trees.

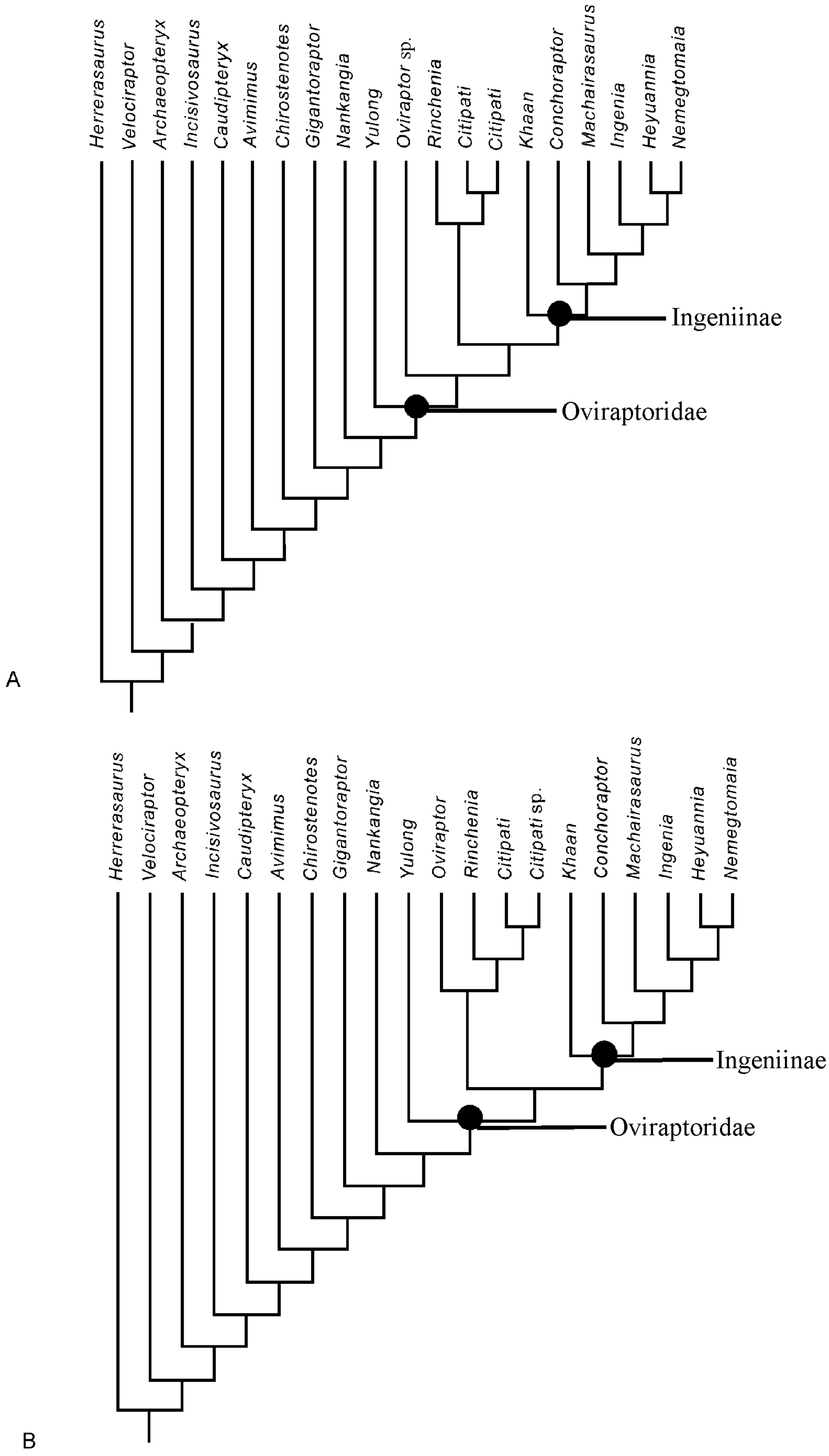

Figure S1. Two most parsimonious trees of 370 steps obtained by a branch and bound search in PAUP, based on a matrix of 20 taxa and 182 characters (CI = 0.58, HI = 0.42, RI = 0.69).

2). Character list

The character list and data matrix are based on [S1], with the addition of *Nankangia*.

1. Ratio of preorbital skull length to basal skull length: 0.60 or more (0); less than 0.60 (1).
2. Pneumatized crestlike prominence on the skull roof: absent (0); present (1).
3. Ratio of width (across premaxilla-maxilla suture) of snout to length: less than 0.3 (0); 0.3–0.4 (1); 0.4 or more (2).
4. Ratio of length of tomial margin of premaxilla to premaxillary height (below external naris): 1.0–1.4 (0); more than 1.7 (1); 0.7 or less (2).
5. Inclination of anteroventral margin of premaxilla relative to horizontally positioned ventral margin of the jugal: vertical (0); posterodorsal (1); anterodorsal (2).
6. Ventral projection of premaxilla below ventral margin of the maxilla: absent (0); small (1); significant (2).
7. Share of premaxilla (ventral) in basal skull length: 0.10 or less (0); 0.12 or more (1).
8. Pneumatization of premaxilla: absent (0); present (1).
9. Ratio of length of maxilla (in lateral view) to basal skull length: 0.4–0.7 (0); less than 0.4 (1).
10. Subantorbital portion of maxilla: not inset medially (0); inset medially (1).
11. Palatal shelf of maxilla with two longitudinal ridges and tooth-like ventral process: absent (0); present (1).
12. Ventral margins of maxilla and jugal: margins form straight line (0); ventral margin of maxilla slopes anteroventrally, its longitudinal axis at an angle of ca. 120° to the longitudinal axis of the jugal (1).
13. Rim around anterior and dorsal margins of antorbital fossa: well pronounced (0); poorly delimited (1).
14. Antorbital fossa: bordered anteriorly by maxilla (0); bordered anteriorly by premaxilla (1).

15. Accessory maxillary fenestrae: absent (0); at least one accessory fenestra present (1).

16. Nasal along midline: longer than frontal (0); shorter than or as long as frontal (1).

17. Nasals: separate (0); fused (1).

18. Subnarial process of nasal: long (0); short (1).

19. Shape of the narial opening: longitudinally oval (0); teardrop-shaped, slightly anteroposteriorly longer than dorsoventrally high (1); anteroposteriorly much longer than dorsoventrally high (2).

20. Nasal recesses: absent (0); present (1).

21. External naris position relative to antorbital fossa: naris and fossa widely separated anteroposteriorly (0); posterior margin of naris reaching level of fossa (1); naris overlaps anterodorsally most of the fossa (2).

22. Anteroventral margin of external naris: at level of maxilla (0); dorsal to maxilla (1).

23. Prefrontal: present (0); absent or fused with lacrimal (1).

24. Lacrimal shaft: not projecting outward beyond orbital plane and lateral surface of the snout (0); medial part of shaft projecting laterally to form flattened transverse bar in front of eye (1).

25. Lacrimal recesses: absent (0); present (1).

26. Ratio of length of orbit to length of antorbital fossa: 0.7–0.9 (0); 1.2 or more (1).

27. Ratio of length of parietal to length of frontal: 0.6 or less (0); 1.0 or more (1).

28. Pneumatization of skull-roof bones: absent (0); present (1).

29. Sagittal crest along interparietal contact: absent (0); present (1).

30. Supratemporal fossa: invading frontal (0); not invading frontal (1).

31. Infratemporal fenestra: dorsoventrally elongate, narrow anterocaudally (0); subquadrate, with anterocaudal length comparable to orbital length (1).

32. Pneumatization of squamosal: absent (0); present (1).

33. Cotylelike incision on ventrolateral margin of the squamosal (for reception of dorsal end of ascending process of the quadratojugal): absent (0); present (1).

34. Ventral ramus of jugal: dorsoventrally deep and lateromedially flattened (0); dorsoventrally shallow or rod like (1).

35. Jugal process of postorbital: not extending ventrally below two-thirds of orbit height (0); long, extending ventrally close to base of postorbital process of jugal (1).

36. Postorbital process of jugal: less than 85° between the postorbital and quadratojugal processes (0) perpendicular to quadratojugal ramus of jugal (1); absent (2).

37. Jugal-postorbital contact: present (0); absent (1).

38. Quadratojugal process of jugal in lateral view: forked (0); not forked (1); fused with quadratojugal (2).

39. Quadratojugal-squamosal contact: absent (0); present (1).

40. Ascending (squamosal) process of quadratojugal: bordering approximately ventral half, or less, of infratemporal fenestra (0); bordering ventral two-thirds or more of infratemporal fenestra (1); absent (2).

41. Angle between ascending and jugal processes of quadratojugal: approximately 90° (0); less than 90° (1).

42. Quadrate process of quadratojugal: well developed, extending posteriorly or posteroventrally beyond posterior margin of ascending process (0); not extending beyond posterior margin of ascending process (1).

43. Dorsal part of quadrate: erect (0); bent backward (1).

44. Otic process of quadrate: articulating only with squamosal (0); articulating with squamosal and lateral wall of braincase (1).

45. Pneumatization of quadrate: absent (0); present (1).

46. Lateral accessory process on distal end of quadrate for contact with quadratojugal: absent (0); present (1).

47. Lateral cotyle for quadratojugal on quadrate: absent (0); present (1).

48. Mandibular condyles of quadrate: posterior to occipital condyle (0); in same vertical plane as occipital condyle (1); anterior to occipital condyle (2).

49. Nuchal transverse crest: pronounced (0); not pronounced (1).

50. Occiput position in relation to ventral margin of jugal-quadratojugal bar: approximately perpendicular (0); inclined anterodorsally (1).

51. Paroccipital process: directed laterally (0); directed ventrally (1).

52. Foramen magnum: smaller than or equal in size to occipital condyle (0); larger than occipital condyle (1).

53. Basal tubera: modestly pronounced (0); well developed, widely separated (1).

54. Pneumatization of basisphenoid: weak or absent (0); extensive (1).

55. Basipterygoid processes: well developed (0); strongly reduced (1); absent (2).

56. Parasphenoid rostrum: horizontal or anterodorsally directed (0); sloping anteroventrally (1).

57. Depression in periotic region: absent (0); present (1).

58. Pneumatization of periotic region: absent or weak (0); extensive (1).

59. Quadrate ramus of pterygoid: distant from braincase wall (0); overlapping braincase (1).

60. Pterygoid basal process for contact with basisphenoid: absent (0); present (1).

61. Ectopterygoid position: lateral to pterygoid (0); anterior to pterygoid (1).

62. Ectopterygoid contacts with maxilla and lacrimal: absent (0); present (1).

63. Ectopterygoid: short anteroposteriorly with hooklike jugal process (0); elongate, shaped like a Viking ship, without hooklike process (1).

64. Massive pterygoid-ectopterygoid longitudinal bar: absent (0); present (1).

65. Palate extending below cheek margin: absent (0); present (1).

66. Palatine: tetraradiate or trapezoid (0); triradiate, without jugal process (1); developed in horizontal, longitudinal, and transverse planes perpendicular to each other (2).

67. Pterygoid wing of palatine: dorsal to pterygoid (0); ventral to pterygoid (1).

68. Maxillary process of palatine: shorter than vomeral process (0); longer than vomeral process (1).

69. Vomer: distant from parasphenoid rostrum (0); approaching or in contact with parasphenoid rostrum (1).

70. Suborbital (ectopterygoid-palatine) fenestra: well developed (0); closed or reduced (1).

71. Jaw joint: distant from midline of skull (0); close to skull midline (1).

72. Movable intramandibular joint: present (0); suppressed (1).

73. Mandibular symphysis: loose (0); tightly sutured (1); fused (2).

74. Extended symphyseal shelf at mandibular symphysis: absent (0); incipient (1); pronounced (2).

75. Downturned symphyseal portion of dentary: absent (0); present (1).

76. U-shaped mandibular symphysis: absent (0); present (1).

77. Ratio of length of retroarticular process to total mandibular length: less than 0.05 or process absent (0); ca. 0.10 (1).

78. Ratio of maximum height of mandible to mandibular length: ca. 0.2 (0); ca. 0.1 (1); 0.3–0.4 (2).

79. Ratio of height to length of external mandibular fenestra: 0.2–0.5 (0); 0.7–1.0 (1); fenestra absent (2).

80. Ratio of length of external mandibular fenestra to total mandibular length: 0.15–0.20 (0); not more than 0.10 or fenestra absent (1); 0.25 or more (2).

81. Process of surangular dividing external mandibular fenestra: absent (0); long (1); short (2).

82. Co-ossification of articular with surangular: absent (0); present (1).

83. Mandibular rami in dorsal view: straight (0); laterally bowed at mid-length (1).

84. Anterodorsal margin of dentary: straight (0); concave (1).

85. Posterior margin of dentary: incised, producing two posterior processes (0); oblique (1).

86. Posterodorsal process of dentary long and shallow: present (0); absent (1).

87. Posteroventral process of dentary shallow and long, extending posteriorly at least to posterior border of external mandibular fenestra: absent (0); present (1).

88. Coronoid eminence: absent (0); present (1).

89. Posterior surangular foramen: present (0); absent (1).

90. Mandibular articular facet for quadrate: comprising surangular and articular (0); formed exclusively of articular (1).

91. Mandibular articular facet for quadrate: with one or two cotyles (0); convex in lateral view, transversely wide (1).

92. Position of articular facet for quadrate: below level of adjoining dorsal margin of mandibular ramus (0); above this margin (1).

93. Anterior part of prearticular: deep, approaching dorsal margin of mandible (0); shallow, straplike, not approaching dorsal mandibular margin (1).

94. Splenial: subtriangular, approaching dorsal mandibular margin (0); straplike, shallow, not approaching margin (1).

95. Mandibular adductor fossa: anteriorly delimited, occupying posterior part of mandible (0); large, anteriorly and dorsally extended, not delimited anteriorly (1).

96. Coronoid bone: well developed (0); strongly reduced (1); absent (2).

97. Premaxillary teeth: present (0); absent (1).

98. Maxillary tooth row: extends at least to level of preorbital bar (0); does not reach level of preorbital bar (1); maxillary teeth absent (2).

99. Dentary teeth: present (0); absent (1).

100. Number of cervicals (excluding cervicodorsal): not more than 10 (0); more than 10 (1).

101. Cranial articular facets of centra in cranial postaxial cervicals: not inclined or only slightly inclined (0); strongly inclined posteroventrally, almost continuous with ventral surfaces of centra (1).

102. Centra of anterior cervicals: not extending posteriorly beyond their respective neural arches (0); extending posteriorly beyond their respective neural arches (1).

103. Epipophyses on postaxial cervicals: in form of a low crest or rugosity (0); prong-shaped (1).

104. Cervical ribs in adults: loosely attached to vertebrae (0); firmly attached (1) or fused (2).

105. Shafts of cervical ribs: longer than their respective centra (0); not longer than their respective centra (1).

106. Pleurocoels on dorsal centra: absent (0); present (1).

107. Ossified uncinate processes on dorsal ribs: absent (0); present (1).

108. Number of vertebrae included in synsacrum in adults: not more than 5 (0); 6 (1); 7–8 (2).

109. Sacral spines in adults: unfused (0); fused (1).

110. Pleurocoels on sacral centra: absent (0); present (1).

111. Transition point on caudals: absent (0); present (1).

112. Number of caudals with transverse processes: 15 or more (0); fewer than 15 (1).

113. Pleurocoels on caudal centra: absent (0); present at least in proximal part of tail (1).

114. Neural spines confined to: at least 23 proximal caudals (0); at most 16 proximal caudals (1).

115. Number of caudals: more than 35 (0); 30 or fewer (1).

116. Distal caudal prezygapophyses: overlapping less than half (0) or at least half (1) of centrum of preceding vertebra.

117. Hypapophyses in cervicodorsal vertebral region: absent (0); small (1); prominent (2).

118. Distal chevrons: deeper than long (0); longer than deep (1).

119. Ratio of length of scapula to length of humerus: 0.8–1.1 (0); 1.2 or more (1); 0.7 or less (2).

120. Acromion: projecting dorsally (0); projecting cranially (1); everted laterally (2).

121. Posteroventral process of coracoid: absent or short, not extending beyond glenoid diameter (0); long, posteroventrally extending beyond glenoid (1).

122. Orientation of glenoid on pectoral girdle: posteroventral (0); lateral (1).

123. Deltopectoral crest: low, its width equal to, or smaller than, shaft diameter (0); expanded, wider than shaft diameter (1).

124. Extent of deltopectoral crest (measured from humeral head to apex): about proximal third of humerus length or less (0); ca. 40%–50% of humerus length (1).

125. Shaft of ulna: straight (0); bowed, convex posteriorly (1).

126. Ratio of length of radius to length of humerus: 0.80 or less (0); 0.85 or more (1). The two bones are almost the same length in 41HIII-0107.

127. Combined lengths of manual phalanges III-1 and III-2: greater than length of phalanx III-3 (0); less than or equal to length of phalanx III-3 (1).

128. Ratio of length of metacarpal I to length of metacarpal II: 0.5 or more (0); less than 0.5 (1).

129. Proximal margin of metacarpal I in dorsal view: straight, horizontal (0); angled due to a medial extent of carpal trochlea (1).

130. Metacarpal II relative to metacarpal III: shorter (0); longer (1); subequal (within 10%) (2).

131. Ratio of length of metacarpal II to length of humerus: 0.4 or less (0); more than 0.4 (1).

132. Ratio of length of manus to length of humerus plus radius: 0.50–0.65 (0); more than 0.65 (1); less than 0.50 (2).

133. Ratio of length of manus to length of femur: 0.3–0.6 (0); more than 0.7 (1).

134. Ratio of length of humerus to length of femur: 0.50–0.69 (0); 0.70 or more (1). Character 134 of Osmólska et al (2004) has been modified so that character state 1 is 0.50 to 0.69 (instead of 0.5 to 0.6).

135. Dorsal margins of opposite iliac blades: well separated from each other (0); close to or contacting each other along their medial sections (1).

136. Dorsal margin of ilium along central portion of blade: straight (0); arched (1).

137. Preacetabular process of ilium relative to postacetabular process (lengths measured from center of acetabulum): shorter or equal (0); longer (1).

138. Preacetabular process: not expanded or weakly expanded ventrally below level of dorsal acetabular margin (0); expanded ventrally well below level of dorsal acetabular margin (1).

139. Morphology of ventral margin of preacetabular process: cuppedicus fossa absent, margin transversely narrow (0); cuppedicus fossa or a wide shelf present (1); margin flat, wide at least close to pubic peduncle (2).

140. Anteroventral extension of preacetabular process: absent (0); with rounded tip (1); hooklike (2).

141. Distal end of postacetabular process: truncated or broadly rounded (0); narrowed or acuminate (1).

142. Anteroposterior length of pubic peduncle: about same as that of ischial peduncle (0); distinctly greater than that of ischial peduncle (1).

143. Dorsoventral extension of pubic peduncle: level with ischial peduncle (0); deeper than ischial peduncle (1).

144. Ratio of length of ilium to length of femur: 0.5–0.79 (0); 0.8 or more (1). (Character modified).

145. Pelvis: propubic (0); mesopubic (1); opisthopubic (2).

146. Pubic shaft: straight (0); concave anteriorly (1).

147. Pubic foot: cranial and posterior processes about equally long (0); anterior process absent or shorter than posterior process (1); anterior process longer than posterior process (2). (0)

148. Posterior margin of ischial shaft: straight, or almost straight (0); distinctly concave (1).

149. Greater trochanter of femur: weakly separated, or not separated, from femoral head (0); distinctly separated from femoral head (1).

150. Anterior and greater trochanters: separated (0); contacting (1).

151. Dorsal extremity of anterior trochanter: well below greater trochanter (0); about level with greater trochanter (1).

152. Fourth trochanter: well developed (0); weakly developed or absent (1).

153. Adductor fossa and associated anteromedial crest on distal femur: weak or absent (0); well developed (1).

154. Distal projection of fibular condyle of femur beyond tibial condyle: absent (0); present (1).

155. Ascending process of astragalus: as tall as it is wide across base (0); taller than wide (1).

156. Distal tarsals: not fused with metatarsus (0); fused with metatarsus (1).

157. Proximal co-ossification of metatarsals II–IV: absent (0); present (1).

158. Arctometatarsus: absent (0); present (1).

159. Length of metatarsal I constituting: more than 50% of metatarsal II length (0); less than 50% of metatarsal II length (1); metatarsal I absent (2).

160. Ratio of maximum length of metatarsus to length of femur: 0.4–0.6 (0); ca. 0.3 (1); 0.7–0.8 (2).

161. Crenulated tomial margin of premaxilla: absent (0); present (1).

162. Frontals flat or weakly arched, not strongly projecting above orbit in lateral view (0) or frontals strongly arched, projecting well above orbit in lateral view to contribute to nasal-frontal crest (1).

163. Exoccipital short, weakly projecting (0) or exoccipital strongly projects ventrally beyond squamosal in lateral view, approaching ventral end of the quadrate (1).

164. Dentary posterodorsal ramus straight or weakly curved (0) or strongly bowed dorsally (1).

165. Dentary symphyseal ventral process absent (0) or prominent symphyseal process present on posteroventral surface of symphysis (1).

166. An arched convexity in the ventral margin of the jaw between the anteroventral margin (chin) and the posteroventral process of the dentary absent (0), or present (1).

167. Lateral surface of dentary smooth (0); bearing a deep fossa, sometimes with associated pneumatopore (1).

168. Angular contributes extensively to the border of the external mandibular fenestra (0); angular largely excluded by surangular (1).

169. Surangular with an anteroposteriorly elongate flange on the ventral edge: absent (0); present (1)

170. External mandibular fenestra elongate (0) or height of external mandibular fenestra subequal to length (1).

171. Dentary contribution to EMF no more than 50 per cent length of dentary (0); exceeds 50 per cent length of dentary (1).

172. Metacarpal I expanded ventrally to cover ventral surface of metacarpal II: absent (0); present (1).

173. Unguals of manual digits II and III: strongly curved (0); weakly curved (1).

174. Manus I-1 slender (0); more robust than II (1); more than 200 per cent diameter of II-1 (2). ORDERED

175. Manual phalanx III-3 longer than phalanx III-2 (0) or length of phalanx III-3 does not exceed length of III-2 (1).

176. Manual phalanx II-2 longer than II-1(0) or manual phalanx II-2 does not exceed the length of preceding phalanx (1).

177. Manual digit II elongate, with combined lengths of manual phalanges II-1 and II-2 subequal to or longer than metacarpal II (0) or combined lengths of II-1 and II-2 subequal to MC II (1) or combined lengths of II-1 and II-2 shorter than MC II (2) ORDERED.

178. Ischium strongly bent posteriorly at midshaft, distal end forms an angle of at least 60º with proximal end: absent (0); present (1).

179. Metatarsus elongate (0); short, length does not exceed 300 per cent of proximal width (1).

180. Ilium tall (0); low and anteroposteriorly elongate, height less than 25 per cent of length (1).

181. Anterior blade of ilium shallower than posterior blade: absent (0); present (1).

182. Calcaneum excludes astragalus from reaching lateral margin of tarsus (0); or small process of astragalus protrudes through a circular opening in edge of calcaneum to reach lateral margin of tarsus (1).

3). Character codings for *Nankangia* gen. et sp. nov.

Characters for phylogenetic analysis and data matrix are adapted from Lü et al. (S1). The complete matrix includes 182 characters and 20 taxa (with *Nankanggia* gen. nov. added).

Nankangia

?????????? ?????????? ?????????? ?????????? ?????????? ?????????? ?????????? ?11101???? ?
??1001??? ????????1? ?????1???1 ??1???1?01 1001?????? ???0111111 1111112110
011?1????? ???11?1??? ???????1?0 00

4). References

S1. Lü JC, Currie PJ, Xu L, Zhang XL, Pu HY, Jia SH (2013) Chicken-sized oviraptorid dinosaurs from central China and their ontogenetic implications. Naturwissenschaften 100: 165–175.

S2. Swofford DL (2002) *Paup**. *Phylogenetic Analysis Using Parsimony* (**and other methods*). Sinauer Associates, Sunderland, Massachusetts.
